# 2-(1-Hydroxypropyn-2-yl)-1-vinylpyrroles: the first successful Favorsky ethynylation of pyrrolecarbaldehydes

**DOI:** 10.3762/bjoc.11.25

**Published:** 2015-02-10

**Authors:** A V Ivanov, V S Shcherbakova, I A Ushakov, L N Sobenina, O V Petrova, A I Mikhaleva, B A Trofimov

**Affiliations:** 1 A. E. Favorsky Irkutsk Institute of Chemistry, Siberian Branch, Russian Academy of Sciences, 1 Favorsky Str., Irkutsk 664033, Russia, Tel: +7 3952 42-56-31; Fax: +7 3952 41-93-46

**Keywords:** acetylene, ethynylation, Favorsky reaction, 1-vinylpyrrole-2-carbaldehyde

## Abstract

1-Vinylpyrrole-2-carbaldehydes react with acetylene at atmospheric pressure in a NaOH/EtOH/DMSO system at 7–10 °C to afford 2-(1-hydroxypropyn-2-yl)-1-vinylpyrroles in 53–94% yield. Thus, the first base-mediated direct ethynylation of pyrrolecarbaldehydes with free acetylene under modified conditions of the Favorsky reaction has been implemented to pave an expedient route to important biomolecules containing a pyrrole ring.

## Introduction

Functionalized pyrroles bearing a terminal acetylenic moiety and hydroxy function as substituents, particularly 2-(1-hydroxypropyn-2-yl)pyrroles, represent important biomolecular intermediates and attractive synthetic building blocks for drug precursors. Currently, they find a growing application in the synthesis of protein kinase inhibitors or modulators [1] and novel cyclin-dependent kinase inhibitors [2]. Such functionalized pyrroles are intermediates for endothelial differentiation gene (EDG-1) receptor antagonists. The latter are effective in preventing and/or treating inflammations, diseases associated with abnormal angiogenesis, cerebral vascular spasm, brain ischemia, cerebral and myocardial infarction, nephritis, immune diseases, and Crohn’s disease [3]. 2-(1-Hydroxypropyn-2-yl)pyrroles have also been employed for the annulation of a cyclopentanone ring onto a pyrrole to form fused bicycles which then have abundant use as synthetic intermediates [4]. Also, 2-(1-hydroxypropyn-2-yl)pyrroles could serve as intermediates for the synthesis of meso-ethynyl-substituted boradiazoindacene (BODIPY) dyes, which have been shown to be potential components of light-harvesting compositions [5].

**Scheme 1:**
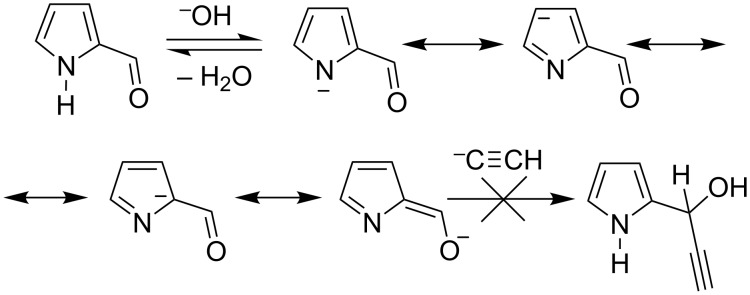
The reaction of pyrrole-2-carbaldehyde with acetylene.

Until now, 2-(1-hydroxypropyn-2-yl)pyrroles have been synthesized exclusively by the addition of ethynylmagnesium halides (Iotsich complexes [6]) to pyrrole-2-carbaldehydes [4,7–8]. Astonishingly, the classic Favorsky ethynylation of pyrrole aldehydes with alkynes in the presence of KOH proves to be absolutely invalid. In fact, our attempt to ethynylate pyrrole-2-carbaldehyde with acetylene under conditions, well suited for the synthesis of secondary acetylenic alcohols (KOH/H_2_O/DMSO, atmospheric pressure, −5 to −7 °C) from aromatic and heteroaromatic aldehydes [9], appeared to be unsuccessful: the starting aldehyde was almost quantitatively recovered. We assumed that the fundamental obstacle in this case is electron delocalization via resonance in the ionized pyrrolecarbaldehyde over the carbonyl function, thus strongly diminishing its electrophilicity (Scheme 1).

If such an assumption was correct, then 1-substituted pyrrole-2-carbaldehydes, incapable of the above ionization, might normally undergo the Favorsky reaction. Indeed, this proves to be true.

This is a concise report on the first successful base-mediated ethynylation of 1-vinylpyrrole-2-carbaldehydes **1a–j** with acetylene.

These *N*-substituted pyrrole-2-carbaldehydes have been chosen for the following reasons: (i) they are easily synthesized in a wide variety from *N*-vinylpyrroles, readily available from ketones (ketoximes) and acetylene [10–13]; (ii) *N*-vinylpyrroles are commonly considered as protected pyrroles [14–20] owing to the easy removal of the *N*-vinyl group; (iii) the vinyl group is an electron-withdrawing substituent relative to the pyrrole ring acting both via inductive and π–π conjugation mechanisms that should increase the electrophilicity of the carbonyl group; (iv) the *N*-vinyl group essentially extends the reactivity and hence potential synthetic utility of the 2-(1-hydroxypropyn-2-yl)pyrroles formed.

## Results and Discussion

After comparative analysis of the available literature data [21–23] and consequent optimization of the reaction conditions we have found that the superbasic catalytic composition NaOH/EtOH/DMSO (the molar ratio 1:1.6:13.6) and a temperature of 7–10 °C (Table 1), which is by ca. ten degrees higher than recommended in the patent [22], are appropriate for the efficient ethynylation of 1-vinylpyrrole-2-carbaldehydes with acetylene. As alkali metal hydroxide for the superbase composition we have chosen NaOH since the more basic KOH was shown to promote a subsequent chalcone formation after ethynylation of 4,5-dihydrobenzo[*g*]indole-2-carbaldehyde (which contains a pyrrole-2-carbaldehyde moiety) with phenylacetylene [21]. As shown on the example of benzaldehyde [22], other ratios of the catalytic composition give inferior yields of the corresponding acetylenic alcohol. In the above optimal superbase composition, ethanol was proved to be a necessary component as it homogenizes the reaction mixture and provides for the controlled decrease of the basicity [23–24]. The latter is needed to prevent the acetylene–allene isomerization of the secondary acetylenic alcohols **2a–j** formed.

**Table 1:** Synthesis of 2-(1-hydroxypropyn-2-yl)-1-vinylpyrroles **2a–j**.

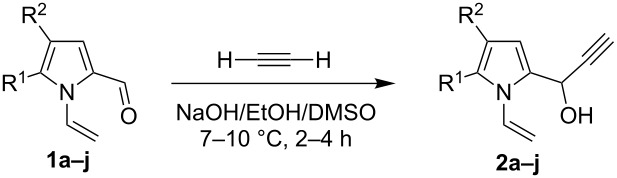				

Entry	1-Vinylpyrrole-2-carbaldehyde **1**	2-(1-Hydroxypropyn-2-yl)-1-vinylpyrrole **2**	*t*, h	Yield, %

a	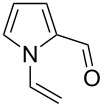	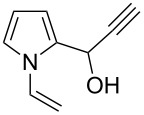	2	68
b	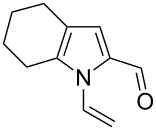	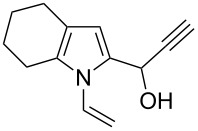	4	63
c	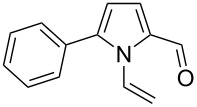	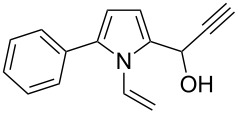	2.5	53
d	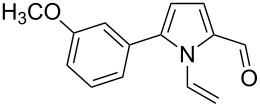	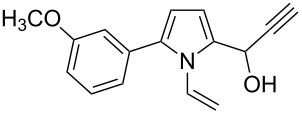	4	94
e	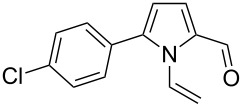	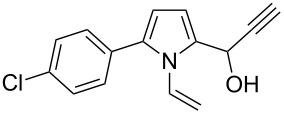	4	67
f	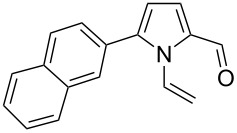	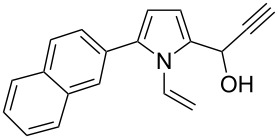	2.5	66
g	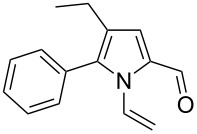	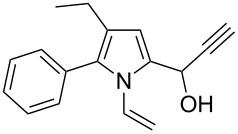	3	55
h	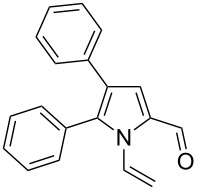	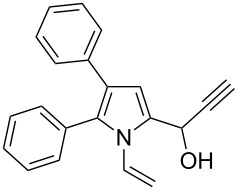	2.5	61
i	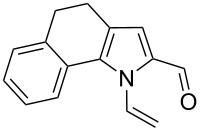	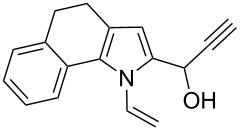	3	60
j	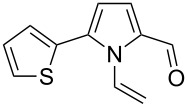	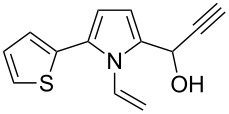	3.5	66

The reaction is carried out at atmospheric pressure (acetylene flow, 7–10 °C, 2–4 h). The yields of hydroxypropynylpyrroles **2a–j** range from 53–94% (Table 1). The reaction was monitored by GLC and was stopped after complete consumption of starting aldehyde. Notably, in GL chromatograms, during the entire course of the reaction, no other peaks except for the starting material and product were discernible. As to the substituents effect on the reaction studied, the variation of yields (mainly 53–68%) is not wide enough to make a reliable conclusion, although the higher yield of acetylenic alcohol **2d** (94%) corresponds to the expectation of the enhanced electron-withdrawing effect of the 3-metoxyphenyl group compared to the unsubstituted phenyl (**1c** → **2c**) that should increase the electrophilicity of the carbonyl function. The range of isolated yields is attributable not only to the difference in the reactivity of aldehydes **1a–j**, but also to the propensity of the product to undergo acetylene–allene isomerization to give the polymerizable vinyl ketones [21]. As seen from Table 1, the reaction tolerates a wide scope of pyrrole-2-carbaldehydes including those possessing aromatic and heteroaromatic substitution at the 5-position, fused aliphatic and aromatic ring systems, and even the 4,5-unsubstituted parent compound **1a**.

**Scheme 2:**
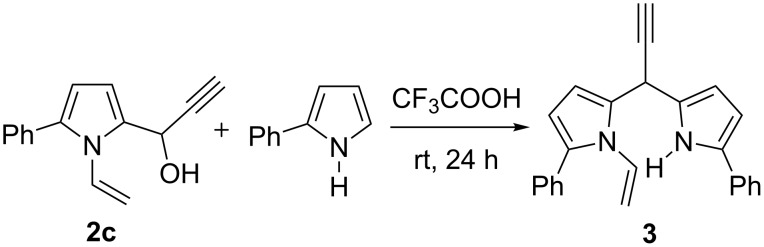
Synthesis of 2-phenyl-5-[1-(5-phenyl-1*H*-pyrrol-2-yl)-2-propynyl]-1-vinylpyrrole (**3**).

Thus, the expedient synthesis of a new family of synthetically and pharmacologically useful functionalized pyrroles has been developed. As an example of a promising application of the synthesized compounds as building blocks we have demonstrated the condensation of acetylenic alcohol **2c** with 2-phenylpyrrole to afford the corresponding dipyrrolomethane **3** in 64% yield (Scheme 2). The reaction readily proceeds at room temperature in the presence of catalytic TFA.

This condensation opens a new pathway to a great variety of synthetically useful, and until now unreported dipyrrolomethanes with acetylenic and *N*-vinyl substituents. Also, it should not be neglected, that 2-(1-hydroxypropyn-2-yl)-1-vinylpyrroles **2** contain the reactive *N*-vinyl group capable of various addition reactions [25–30] and polymerization [31–33], that remarkably extends their potential applications.

## Conclusion

In conclusion, the base-mediated Favorsky direct ethynylation of pyrrolecarbaldehydes with free acetylene has been successfully accomplished thus introducing into the pyrrole chemistry a new family of highly potent building blocks and precursors of light-harvesters and drugs. The fundamental reason of our failures to ethynylate unsubstituted pyrrolecarbaldehydes under the Favorsky conditions has been shown to be the electron delocalization via resonance from the ionized NH moiety to the aldehyde function. Thus, the long-year siege of the pyrrolecarbaldehyde fortress has been victoriously ended.

## Supporting Information

File 1 Experimental and analytical data.

## References

[1] Luk K-C, Mahaney P E, Mischke S G (2001). 4- and 5-alkynyloxindoles and 4- and 5-alkenyloxindoles. U.S. Patent.

[2] Luk K-C, Mahaney P E, Mischke S G (2000). 4- and 5-alkynyloxindoles and 4- and 5-alkenyloxindoles. W.O. Patent.

[3] Sato S, Nakamura T, Nara F, Komesu K (2005). Preparation of arylalkyne derivatives having EDG receptor antagonist effect. Japanese Pat..

[4] Yamabe H, Mizuno A, Kusama H, Iwasawa N (2005). J Am Chem Soc.

[5] Kolemen S, Cakmak Y, Ozdemir T, Erten-Ela S, Buyuktemiz M, Dede Y, Akkaya E U (2014). Tetrahedron.

[6] Silverman G S, Rakita P E Handbook of Grignard reagents.

[7] Liu J-J, Konzelmann F, Luk K-C (2003). Tetrahedron Lett.

[8] Nobuhiro J, Hirayama M, Choshi T, Kamoshita K, Maruyama S, Sukenaga Y, Ishizu T, Fujioka H, Hibino S (2006). Heterocycles.

[9] Sobenina L N, Tomilin D N, Petrova O V, Mikhaleva A I, Trofimov B A (2013). Russ J Org Chem.

[10] Abele E, Lucevics E (2000). Heterocycles.

[11] Mikhaleva A I, Schmidt E Yu, Kartsev V G (2002). Selected methods for synthesis and modification of heterocycles.

[12] Tedeschi R J, Meyers R A (2004). Acetylene. Encyclopedia of Physical Science and Technology.

[13] Wang Z (2009). Comprehensive Organic Name Reactions and Reagents.

[14] Trofimov B A, Korostova S E, Mikhaleva A I, Sobenina L N, Vasil’ev A N (1982). Chem Heterocycl Compd.

[15] Gonzalez C, Greenhouse R, Tallabs R, Muchowski J M (1983). Can J Chem.

[16] Trofimov B A, Korostova S E, Shevchenko S G, Mikhaleva A I, Matel’ N L (1996). Russ J Org Chem.

[17] Varlamov A V, Voskresenskii L V, Borisova T N, Chernyshev A I, Levov A N (1999). Chem Heterocycl Compd.

[18] Borisova T N, Bonifas N, Voskresenskii L G, Chernyshev A I, Varlamov A V, Krapivko A P (2004). Chem Heterocycl Compd.

[19] Petrushenko I K, Smirnov V I, Petrushenko K B, Schmidt E Yu, Zorina N V, Rusakov Yu Yu, Vasil’tsov A M, Mikhaleva A I, Trofimov B A (2007). Russ J Gen Chem.

[20] Schmidt E Yu, Trofimov B A, Mikhaleva A I, Zorina N V, Protsuk N I, Petrushenko K B, Ushakov I A, Dvorko M Yu, Méallet-Renault R, Clavier G (2009). Chem – Eur J.

[21] Schmidt E Yu, Bidusenko I A, Protzuk N I, Ushakov I A, Ivanov A V, Mikhaleva A I, Trofimov B A (2012). Chem Heterocycl Compd.

[22] Trofimov B A, Petrova O V, Sobenina L N, Mikhaleva A I (2014). Method of producing 1-phenyl propargyl alcohol. Russian Patent.

[23] Buncel E, Wilson H, Gold V, Bethell D (1977). Advances in physical organic chemistry.

[24] Trofimov B A, Potekhin A A, Kostikov R R, Baird M S (2004). Superbase catalysts and reagents: the concept, application, perspectives. Modern problems of organic chemistry.

[25] Schmidt E Yu, Cherimichkina N A, Bidusenko I A, Protzuk N I, Trofimov B A (2014). Eur J Org Chem.

[26] Trofimov B A, Ivanov A V, Ushakov I A, Schmidt E Yu, Sobenina L N, Vasil’tsov A M, Mikhaleva A I (2012). Dalton Trans.

[27] Dmitrichenko M Yu, Ivanov A V, Bidusenko I A, Ushakov I A, Mikhaleva A I, Trofimov B A (2011). Tetrahedron Lett.

[28] Sobenina L N, Mikhaleva A I, Sergeeva M P (1992). Sulfur Lett.

[29] Trofimov B A, Malysheva S F, Suchov B G, Belogorlova N A, Schmidt E Yu, Sobenina L N, Kuimov V A, Gusarova N K (2003). Tetrahedron Lett.

[30] Trofimov B A, Gusarova N K, Sukhov B G, Malysheva S F, Tarasova O A, Belogorlova N A, Maximova M A, Tunik S P (2005). Synthesis.

[31] Skotheim T, Lundstrom I, Prejra J (1981). J Electrochem Soc.

[32] Sessler J L, Roznyatovskiy V, Dan Pantos G, Borisova N E, Reshetova M D, Lynch V M, Krustalev V N, Ustynyuk J A (2005). Org Lett.

[33] Furuta H, Maeda H, Furuta T, Osuka A (2000). Org Lett.

