# Supplementary material for: 2-(1-Hydroxypropyn-2-yl)-1-vinylpyrroles: the first successful Favorsky ethynylation of pyrrolecarbaldehydes
Source: Beilstein J Org Chem. 2015 Feb 10;11:228–32. doi: 10.3762/bjoc.11.25 (PMC4362022; doi:10.3762/bjoc.11.25)
Supplement: File 1 — Experimental and analytical data. [file Beilstein_J_Org_Chem-11-228-s001.pdf]

Supporting Information

for

2-(1-Hydroxypropyn-2-yl)-1-vinylpyrroles: the first successful Favorsky ethynylation of pyrrolecarbaldehydes

A. V. Ivanov, V. S. Shcherbakova, I. A. Ushakov, L. N. Sobenina, O. V. Petrova, A. I. Mikhaleva, B. A. Trofimov*

Address: A. E. Favorsky Irkutsk Institute of Chemistry, Siberian Branch, Russian Academy of Sciences, 1 Favorsky Str., Irkutsk 664033 Russia, Tel: +7 3952 42-56-31; Fax: +7 3952 41-93-46.

Email: B. A. Trofimov * - boris_trofimov@irioch.irk.ru

*Corresponding author

Experimental and analytical data

Experimental

General Description

^{1}H (400.13 MHz) and ^{13}C (100.6 MHz) NMR spectra were recorded on a "Bruker Avance 400" instrument in $CDCl_3$. The ^{1}H and ^{13}C chemical shifts were referenced to HMDS. Elemental analysis (C, H, N) was performed on an EA FLASH 1112 Series (CHN Analyzer). EtOH, NaOH, DMSO (content of water 0.2–0.3%) and all other chemicals and solvents are commercially available and were used without further purification. 1-Vinylpyrrole-2-carbaldehydes **1** were prepared according to [Mikhaleva, A. I.; Ivanov, A. V.; Skital'tseva, E. V.; Ushakov, I. A.; Vasil'tsov, A. M.; Trofimov, B. A. *Synthesis* **2009**, *4*, 587−590]. Aldehydes **1e,g,h** were synthesized for the first time.

Synthesis of 2-(1-hydroxypropyn-2-yl)-1-vinylpyrrole (2a).

In a flask equipped with magnetic stirrer and reflux condenser DMSO (14 mL), powdered NaOH (0.57 g, 14 mmol) and EtOH (1.4 mL, 10% v/v of DMSO) were placed. The mixture was heated up to 125–130 °C, and then acetylene was passed at atmospheric pressure, simultaneously cooling to 7–10 °C. Then a solution of 1-vinylpyrrole-2-carbaldehyde (**1a**, 1.75 g, 14 mmol) in DMSO (3 mL) was added dropwise and the reaction mixture was stirred under acetylene flow (subsurface sparge) at the same temperature for 1.5 h. The reaction was monitored by GLC. After completion of the reaction the mixture was diluted with a solution of NH_4Cl (1.51 g, 28 mmol) in H_2O (50 mL) and it was extracted with diethyl ether (7 × 15 mL). The organic layer was washed with water (5 × 15 mL) and dried overnight over $MgSO_4$. After removal of the solvent the crude product was purified by column chromatography (SiO_2, hexane−diethyl ether, 2:1 v/v) to give 1.4 g (68%) of 1-(1-vinylpyrrol-2-yl)-2-propyn-1-ol (**2a**) as yellow oil. ^{1}H NMR (400.13 MHz, $CDCl_3$) δ 7.25 (dd, J = 15.7, 8.8 Hz, 1 H, H_X),

7.08 (dd, J = 3.2, 1.9 Hz, 1 H, H5), 6.41 (dd, J = 3.5, 1.9 Hz, 1 H, H3), 6.17 (dd, J = 3.5, 3.2 Hz, 1 H, H4), 5.54 (dd, J = 7.0, 2.3 Hz, 1 H, CH), 5.18 (dd, J = 15.7, 1.3 Hz, 1 H, H_B), 4.77 (dd, J = 8.8, 1.3 Hz, 1 H, H_A), 2.66 (d, J = 2.3 Hz, 1 H, ≡CH), 2.28 (br. s, 1 H, OH); ^{13}C NMR (100.6 MHz, $CDCl_3$) δ 130.9 (Cα), 130.4 (C2), 119.0 (C5), 110.4 (C3), 109.1 (C4), 99.2 (Cβ), 82.1 (<u>C</u>≡CH), 74.1 (≡CH), 57.2 (COH). ν_{max} (KBr) 3367 (OH), 3291, 2120 (≡C−H), 1643 (N−CH=CH_2) cm^{-1}. Anal. Calcd (%) for C_9H_9NO: C, 73.45; H, 6.16; N, 9.52. Found: C, 73.23; H, 6.16; N, 9.50. 2-(1-Hydroxypropyn-2-yl)-1-vinylpyrroles **2b–j** were prepared analogously.

1-(1-Vinyl-4,5,6,7-tetrahydroindol-2-yl)-2-propyn-1-ol (**2b**): from 2.2 g of carbaldehyde **1b** 1.6 g (63%) of propyn-1-ol **2b** was obtained as yellow oil. ^{1}H NMR (400.13 MHz, $CDCl_3$) δ 6.96 (dd, J = 16.0, 9.1 Hz, 1 H, H_X), 6.24 (s, 1 H, H3), 5.45 (br. d, J = 2.3 Hz, 1 H, CH), 5.16 (d, J = 16.0 Hz, 1 H, H_B), 4.90 (d, J = 9.1 Hz, 1 H, H_A), 2.59–2.57 (m, 2 H, 7-CH_2), 2.57 (d, J = 2.3 Hz, 1 H, ≡CH), 2.46–2.43 (m, 2 H, 4-CH_2), 2.01 (br. s, 1 H, OH), 1.78–1.65 (m, 4 H, 5-CH_2 , 6-CH_2); ^{13}C NMR (100.6 MHz, $CDCl_3$) δ 130.4 (C2), 130.2 (Cα), 129.5 (C7a), 118.45 (C4a), 109.5 (Cβ), 104.8 (C3), 82.6 (<u>C</u>≡CH), 73.7 (≡CH), 57.5 (COH), 24.1, 23.6 23.2, 23.0 (CH_2). ν_{max} (KBr) 3405 (OH), 3287, 2120 (≡C−H), 1643 (N−CH=CH_2) cm^{-1}. Anal. Calcd (%) for $C_{13}H_{15}NO$: C, 77.58; H, 7.51; N, 6.96. Found: C, 74.30; H, 7.62; N, 6.95.

1-(5-Phenyl-1-vinylpyrrol-2-yl)-2-propyn-1-ol (**2c**)**:** from 3 g of carbaldehyde **1c** 1.8 g (53%) of propyn-1-ol **2c** was obtained as yellow oil. ^{1}H NMR (400.13 MHz, $CDCl_3$) δ 7.40–7.37 (m, 2 H, Ho), 7.35–7.32 (m, 2 H, Hm), 7.28–7.25 (m, 1 H, Hp), 6.83 (dd, J = 15.9, 8.9 Hz, 1 H, H_X), 6.56 (d, J = 3.8 Hz, 1 H, H3), 6.20 (d, J = 3.8 Hz, 1 H, H4), 5.50 (br. d, J = 5.0 Hz, 1 H, CH), 5.28 (d, J = 15.9 Hz, 1 H, H_B), 5.04 (dd, J = 8.9 Hz, 1 H, H_A), 2.61 (d, J = 2.4 Hz, 1 H, ≡CH), 2.22 (d, J = 5.0 Hz, 1 H, OH). ^{13}C NMR (100.6 MHz,

$CDCl_3$) δ 136.2 (C5), 132.9 (Ci), 132.7 (C2), 131.3 (Cα), 129.0 (Co), 128.3 (Cm), 127.2 (Cp), 110.5 (C3), 110.0 (Cβ), 109.4 (C4), 82.6 (<u>C</u>≡CH), 73.8 (≡CH), 57.4 (COH). ν_{max} (KBr): 3376 (OH), 3290, 2120 (≡C−H), 1643 (N−CH=CH_2) cm^{-1}. Anal. Calcd (%) for $C_{15}H_{13}NO$: C, 80.69; H, 5.87; N, 6.27. Found: C, 80.37; H, 6.24; N, 5.93.

1-[5-(3-Methoxyphenyl)-1-vinylpyrrol-2-yl]-2-propyn-1-ol (**2d**): from 1.2 g of carbaldehyde **1d** 1.26 g (94%) of propyn-1-ol **2d** was obtained as brown oil. Yield: 1.26 g (94%). ^{1}H NMR (400.13 MHz, $CDCl_3$) δ 7.30 (dd, J = 8.0, 7.8 Hz, 1 H, H5 ar), 7.04–7.01 (m, 1 H, H6 ar), 6.98 (dd, J = 2.4, 1.7 Hz, 1 H, H2 ar), 6.89 (dd, J = 15.9, 8.8 Hz, 1 H, H_X), 6.88–6.85 (m, 1 H, H4 ar), 6.61 (d, J = 3.7 Hz, 1 H, H3), 6.27 (d, J = 3.7 Hz, 1 H, H4), 5.55 (dd, J = 6.1, 2.2 Hz, 1 H, CH), 5.36 (d, J = 15.9 Hz, 1 H, H_B), 5.11 (d, J = 8.8 Hz, 1 H, H_A), 3.83 (s, 3 H, OCH_3), 2.67 (d, J = 2.2 Hz, 1 H, ≡CH), 2.32 (d, J = 6.1 Hz, 1 H, OH). ^{13}C NMR (100.6 MHz, $CDCl_3$) δ 159.4 (C3 ar), 136.0 (C5), 134.2 (C1 ar), 132.7 (C2), 131.4 (Cα), 129.4 (C5 ar), 121.6 (C6 ar), 114.6 (C2 ar), 112.9 (C4 ar), 110.6 (C3), 110.0 (Cβ), 109.5 (C4), 82.6 (<u>C</u>≡CH), 73.9 (≡CH), 57.4 (COH), 55.3 (OCH_3). ν_{max} (KBr): 3418 (OH), 3288, 2120 (≡C−H), 1643 (N−CH=CH_2) cm^{-1}. Anal. Calcd (%) for $C_{16}H_{15}NO_2$: C, 75.87; H, 5.97; N, 5.53. Found: C, 75.57; H, 5.80; N, 5.33.

5-(4-chlorophenyl)-1-vinylpyrrole-2-carbaldehyde (**1e**): Yield 65.1%, brown oil. ^{1}H NMR (400.13 MHz, $CDCl_3$) δ 9.57 (s,1 H, CHO), 7.35 (dd, J = 15.9, 8.8 Hz, 1 H, H_X) 7.34–7.30 (m, 4 H, Har), 7.00 (d, J = 3.9 Hz, 1 H, H3), 6.31 (d, J = 3.9 Hz, 1 H, H4), 5.07 (d, J = 8.8 Hz, 1 H, H_A), 4.82 (d, J = 15.9 Hz, 1 H, H_B). ^{13}C NMR (100.6 MHz, $CDCl_3$) δ 179.4 (CHO), 140.8 (C5), 134.4 (CCl), 133.5 (C2), 131.0 (Cα), 130.4, 129.8, 128.7 (Car), 124.2 (C3), 112.9 (Cβ), 112.7 (C4). Anal. Calcd (%) for $C_{13}H_{10}ClNO$: C, 67.40; H, 4.35; Cl, 15.30; N, 6.05. Found: C, 67.45; H, 4.40; Cl, 15.25; N, 6.00.

1-[5-(4-Chlorophenyl)-1-vinylpyrrol-2-yl]-2-propyn-1-ol (**2e**): from 1.5 g of carbaldehyde **1e** 1.25 g (67%) of propyn-1-ol **2e** was obtained as brown oil. ^{1}H NMR (400.13 MHz, $CDCl_3$) *δ* 7.33 (m, 4 H, Har), 6.84 (dd, *J* = 16.0, 8.8 Hz, 1 H, H_X), 6.57 (d, *J* = 3.7 Hz, 1 H, H3), 6.22 (d, *J* = 3.7 Hz, 1 H, H4), 5.51 (br. s, 1 H, CH), 5.28 (d, *J* = 16.0 Hz, 1 H, H_B), 5.10 (d, *J* = 8.8 Hz, 1 H, H_A), 2.65 (d, *J* = 2.3 Hz, 1 H, ≡CH), 2.26 (br. s, 1 H, OH). ^{13}C NMR (100.6 MHz, $CDCl_3$) *δ* 134.7 (C5), 133.2 (CCl), 133.0 (Ci), 131.4 (C2), 131.1 (Cα), 130.1 (Co), 128.6 (Cm), 110.6 (C3), 110.7 (Cβ), 109.8 (C4), 82.5 (<u>C</u>≡CH), 74.0 (≡CH), 57.3 (COH). ν_{max} (KBr): 3376 (OH), 3290, 2120 (≡C−H), 1643 (N−CH=CH_2) cm^{-1}. ν_{max} (KBr): 3347 (OH), 3298, 2121 (≡C−H), 1643 (N−CH=CH_2) cm^{-1}. Anal. Calcd (%) for $C_{15}H_{12}ClNO$: C, 69.91; H, 4.69; Cl, 13.76; N, 5.44. Found: C, 69.95; H, 4.75; Cl, 13.55; N, 5.35.

1-[5-(2-Naphthyl)-1-vinylpyrrol-2-yl]-2-propyn-1-ol (**2f**): from 1.5 g of carbaldehyde **1f** 1.09 g (66%) of propyn-1-ol **2f** was obtained as brown oil. ^{1}H NMR (400.13 MHz, $CDCl_3$) *δ* 7.90–7.84 (m, 4 H, H3 naph, H4 naph, H7 naph, H8 naph), 7.51–7.47 (m, 3 H, H1 naph, H5 naph, H6 naph), 6.96 (dd, *J* = 15.9, 8.8 Hz, 1 H, Hx), 6.67 (d, *J* = 3.7 Hz, 1 H, H3), 6.38 (d, *J* = 3.7 Hz, 1 H, H4), 5.59 (dd, *J* = 7.0, 2.3 Hz, 1 H, CH), 5.36 (d, *J* = 15.9 Hz, 1 H, H_B), 5.12 (d, *J* = 8.8 Hz, 1 H, H_A), 2.70 (d, *J* = 2.3 Hz, 1 H, ≡CH), 2.29 (d, *J* = 7.0 Hz, 1 H, OH). ^{13}C NMR (100.6 MHz, $CDCl_3$) *δ* 136.1 (C5), 133.4 (C3a naph), 132.9 (C2), 132.5 (C7a naph), 131.4 (Cα), 130.4 (C2 naph), 128.1 (C8 naph), 127.9 (C4 naph, C7 naph),127.6 (C3 naph), 127.2 (C1 naph), 126.4 (C6 naph), 126.1 (C5 naph), 110.7 (C3), 110.3 (Cβ), 109.9 (C4), 81.6 (<u>C</u>≡CH), 74.0 (≡CH), 57.5 (COH). ν_{max} (KBr): 3385 (OH), 3292, 2120 (≡C−H), 1642 (N−CH=CH_2) cm^{-1}. Anal. Calcd (%) for $C_{19}H_{15}NO$: C, 83.49; H, 5.53; N, 5.12. Found: C, 83.09; H, 5.23; N, 4.95.

4-ethyl-5-phenyl-1-vinylpyrrole-2-carbaldehyde (**1g**): Yield 49.6%, light brown oil. ^{1}H NMR (400.13 MHz, $CDCl_3$) δ 9.58 (s,1 H, CHO), 7.42–7.24 (m, 5 H, Har), 7.29 (dd, J = 15.8, 8.8 Hz, 1 H, H_X), 6.96 (s, 1 H, H3), 4.85 (d, J = 8.8 Hz, 1 H, H_A), 4.64 (d, J = 15.8 Hz, 1 H, H_B), 2.36 (q, J = 7.5 Hz, 2 H, CH_2), 1.09 (t, J = 7.5 Hz, 3 H, CH_3). ^{13}C NMR (100.6 MHz, $CDCl_3$) δ 178.8 (CHO), 138.5 (C5), 131.4 (C2), 130.9 (Ci), 130.8 (Cα), 130.0 (Co), 128.3 (Cm), 128.2 (Cp), 127.6 (C4), 123.4 (C3), 109.8 (Cβ), 18.7 (CH_2), 14.9 (CH_3). Anal. Calcd (%) for $C_{15}H_{15}NO$: C, 79.97; H, 6.71; N, 6.22. Found: C, 79.77; H, 6.52; N, 6.03.

1-(4-Ethyl-5-phenyl-1-vinylpyrrol-2-yl)-2-propyn-1-ol (**2g**): from 1.35 g of carbaldehyde **1g** 0.83 g (55%) of propyn-1-ol **2g** was obtained as brown oil. ^{1}H NMR (400.13 MHz, $CDCl_3$) δ 7.36 (m, 2 H, Hm), 7.29 (m, 1 H, Hp), 7.26 (m, 2 H, Ho), 6.71 (dd, J = 15.9, 8.9 Hz, 1 H, H_X), 6.51 (s, 1 H, H3), 5.50 (br. d, J = 2.4 Hz, 1 H, CH), 5.07 (d, J = 15.9 Hz, 1 H, H_B), 4.83 (d, J = 8.9 Hz, 1 H, H_A), 2.62 (d, J = 2.4 Hz, 1 H, ≡CH), 2.38 (q, J = 7.6 Hz, 2 H, CH_2), 1.18 (t, J = 7.6 Hz, 3 H, CH_3). ^{13}C NMR (100.6 MHz, $CDCl_3$) δ 132.4 (Ci), 131.5 (C5), 131.1 (Cα), 130.8 (C2), 130.7 (Co), 128.2 (Cm), 127.3 (Cp), 124.6 (C4), 110.5 (C3), 107.6 (Cβ), 82.8 ($\underline{C}$≡CH), 73.8 (≡CH), 57.3 (COH), 19.5 (CH_2), 15.5 (CH_3). ν_{max} (KBr): 3397 (OH), 3292, 2120 (≡C−H), 1641 (N−CH=CH_2) cm^{-1}. Anal. Calcd (%) for $C_{17}H_{17}NO$: C, 81.24; H, 6.82; N, 5.57. Found: C, 81.04; H, 6.90; N, 5.72.

4,5-diphenyl-1-vinylpyrrole-2-carbaldehyde (**1h**): Yield 70.0%, coloress oil. ^{1}H NMR (400.13 MHz, $CDCl_3$) δ 9.68 (s,1 H, CHO), 7.40–7.10 (m, 12 H, ar, H3, H_X), 5.02 (d, J = 8.8 Hz, 1 H, H_A), 4.78 (d, J = 16.0 Hz, 1 H, H_B). ^{13}C NMR (100.6 MHz, $CDCl_3$) δ 179.4 (CHO), 138.3 (C5), 134.1 (Ci), 132.1 (C2), 131.0 (Cα), 130.9, 128.7, 128.3, 128.2, 126.5, (Car), 126.2 (C4), 123.6 (C3), 112.2 (Cβ). Anal. Calcd (%) for $C_{19}H_{15}NO$: C, 83.49; H, 5.53; N, 5.12. Found: C, 83.31; H, 5.52; N, 5.03.

1-(4,5-Diphenyl-1-vinylpyrrol-2-yl) -2-propyn-1-ol (**2h**): from 1.3 g of carbaldehyde **1h** 0.87 g (61%) of propyn-1-ol **2h** was obtained as red crystals. Mp 56 ºC. ^{1}H NMR (400.13 MHz, $CDCl_3$) *δ* 7.40–7.35 (m, 2 H, Har), 7.32–7.30 (m, 3 H, Har), 7.20–7.15 (m, 5 H, Har), 6.85 (s, 1 H, H3), 6.76 (dd, *J* = 16.0, 8.9 Hz, 1 H, H_X), 5.62 (br. d, *J* = 2.0 Hz, 1 H, CH), 5.28 (d, *J* = 16.0 Hz, 1 H, H_B), 5.02 (d, *J* = 8.9 Hz, 1 H, H_A), 2.71 (d, *J* = 2.3 Hz, 1 H, ≡CH), 2.31 (br. s, 1 H, OH). ^{13}C NMR (100.6 MHz, $CDCl_3$) *δ* 135.6 (C5), 131.7, 131.6, 131.4, 128.5, 128.2, 128.14, 127.9, 125.7 (Car), 132.3 (C2), 131.0 (Cα), 130.7 (Co), 123.1 (C4), 111.1 (C3), 109.6 (Cβ), 82.6 (<u>C</u>≡CH), 74.0 (≡CH), 57.4 (COH). ν_{max} (KBr): 3429 (OH), 3288, 2119 (≡C−H), 1640 (N−CH=CH_2) cm^{-1}. Anal. Calcd (%) for $C_{21}H_{17}NO$: C, 84.25; H, 5.72; N, 4.68. Found: C, 84.15; H, 5.94; N, 4.55.

1-(1-Vinyl-4,5-dihydrobenzo[*g*]indol-2-yl)-2-propyn-1-ol (**2i**): from 1.5 g of carbaldehyde **1i** 1 g (60%) of propyn-1-ol **2i** was obtained as orange oil. ^{1}H NMR (400.13 MHz, $CDCl_3$) *δ* 7.53–7.50 (m, 1 H, H9), 7.18–7.15 (m, 1 H, H6), 7.14–7.11 (m, 1 H, H8), 7.04–7.01 (m, 1 H, H7), 7.02 (dd, *J* = 15.8, 8.5 Hz, 1 H, H_X), 6.41 (s, 1 H, H3), 5.53 (d, *J* = 15.8 Hz, 1 H, H_B), 5.43 (d, *J* = 2.3 Hz, 1 H, CH), 5.24 (d, *J* = 8.5 Hz, 1 H, H_A), 2.83–2.80 (m, 2 H, H5), 2.59–2.54 (m, 2 H, H4), 2.58 (d, *J* = 2.3 Hz, 1 H, ≡CH), 2.29 (br. s, 1 H, OH). ^{13}C NMR (100.6 MHz, $CDCl_3$) *δ* 136.4 (C9a), 132.9 (C2), 132.2 (Cα), 130.2 (C9b), 129.3 (C5a), 128.3 (C6), 126.2 (C8), 125.3 (C7), 122.7 (C4a), 121.8 (C9), 111.4 (Cβ), 108.9 (C3), 82.7 (<u>C</u>≡CH), 73.7 (≡CH), 57.0 (COH), 30.7 (C5), 22.1 (C4). ν_{max} (KBr): 3369 (OH), 3300, 2246 (≡C−H), 1639 (N−CH=CH_2) cm^{-1}. Anal. Calcd (%) for $C_{17}H_{15}NO$: C, 81.90; H, 6.06; N, 5.62. Found: C, 81.60; H, 6.25; N, 5.33.

1-[5-(2-Thienyl)-1-vinylpyrrol-2-yl]-2-propyn-1-ol (**2j**): from 1.6 g of carbaldehyde **1j** 1.2 g (66%) of propyn-1-ol **2j** was obtained as yellow oil. ^{1}H NMR (400.13 MHz, $CDCl_3$) *δ* 7.30

(dd, J = 5.0, 1.4 Hz, 1 H, H5’), 7.08 (dd, J = 3.6, 1.4 Hz, 1 H, H3’), 7.06 (dd, J = 5.0, 3.6 Hz, 1 H, H4’), 6.92 (dd, J = 15.8, 8.7 Hz, 1 H, H_X), 6.59 (d, J = 3.7 Hz, 1 H, H3), 6.33 (d, J = 3.7 Hz, 1 H, H4), 5.52 (d, J = 15.8 Hz, 1 H, H_B), 5.52 (d, J = 2.3 Hz, 1 H, CH), 5.21 (d, J = 8.7 Hz, 1 H, H_A), 2.66 (d, J = 2.3 Hz, 1 H, ≡CH), 2.19 (br. s, 1 H, OH). ^{13}C NMR (100.6 MHz, $CDCl_3$) δ 134.4 (C2’), 133.0 (C2), 131.1 (Cα), 128.9 (C5), 127.3 (C4’), 126.4 (C3’), 125.4 (C5’), 111.4 (Cβ), 110.6 (C3), 110.2 (C4), 82.5 (<u>C</u>≡CH), 73.9 (≡CH), 57.3 (COH). ν_{max} (KBr): 3418 (OH), 3289, 2120 (≡C−H), 1642 (N−CH=CH_2) cm^{-1}. Anal. Calcd (%) for $C_{13}H_{11}NOS$: C, 68.10; H, 4.84; N, 6.11; S 13.98. Found: C, 67.79; H, 4.76; N, 6.01; S, 13.68.

Synthesis of 2-phenyl-5-[1-(5-phenyl-1*H*-pyrrol-2-yl)-2-propynyl]-1-vinylpyrrole (3). The CF_3COOH (0.02-0.03 mL) was added to the mixture of 1-(5-phenyl-1-vinylpyrrol-2-yl)-2-propyn-1-ol (**2c**) (0.2 g, 0.89 mmol) and 2-phenylpyrrole (0.13 g, 0.89 mmol) in dried dichloromethane (10 mL) and the mixture was stirred at room temperature for 24 h. Solution of $NaHCO_3$ (0.15 g, 1.78 mmol) in H_2O (10 mL) was added and the mixture was stirred at room temperature for 1 h, then extracted with diethyl ether (3 × 5 mL). The organic layer was washed with water (3 × 5 mL) and dried overnight over K_2CO_3. The residue after removal of the solvent was purified by column chromatography (SiO_2, hexane–diethyl ether, 2:1 v/v) to give 0.20 g (64%) of 2-phenyl-5-[1-(5-phenylpyrrol-2-yl)-2-propynyl]-1-vinylpyrrole (**3**) as brown solid. ^{1}H NMR (400.13 MHz, $CDCl_3$) δ 8.43 (br. s, 1 H, NH), 7.48−7.44 (m, 4 H, Ho, Ho’), 7.38−7.28 (m, 6 H, Hm, Hm’, Hp, Hp’), 6.83 (dd, J = 15.9, 8.6 Hz, 1 H, H_X), 6.48 (dd, J = 3.7, 2.7 Hz, 1 H, H4’), 6.33 (d, J = 3.5 Hz, 1 H, H4), 6.29 (d, J = 3.5 Hz, 1 H, H3), 6.20 (dd, J = 3.7, 2.5 Hz, 1 H, H3’), 5.33 (d, J = 2.5 Hz, 1 H, CH), 5.06 (d, J = 8.6 Hz, 1 H, H_A), 5.05 (d, J = 15.9 Hz, 1 H, H_B), 2.50 (d, J = 2.5 Hz, 1 H, ≡CH). ^{13}C NMR (100.6 MHz, $CDCl_3$) δ 135.2 (C2), 133.3 (Ci’), 132.7 (Ci), 131.9 (C5’), 131.4 (Cα), 131.3 (C5), 129.4 (C2’), 128.9 (Co, Cm’), 128.3 (Cm),

126.9 (Cp), 126.2 (Cp’), 123.8 (Co’), 111.3 (Cβ), 109.8 (C3), 109.6 (C4), 108.6 (C3’), 106.5 (C4’), 82.2 (C≡CH), 71.8 (≡CH), 29.9 (CH). v_{max} (KBr): 3435 (NH), 2856 (CH), 2244 (≡C−H), 1642 (N−CH=CH_2) cm^{-1}. Anal. Calcd (%) for $C_{25}H_{20}N_2$: C, 86.18; H, 5.79; N, 8.04. Found: C, 86.28; H, 5.85; N, 8.14.
